# Repeated sampling facilitates within- and between-subject modeling of the human sperm transcriptome to identify dynamic and stress-responsive sncRNAs

**DOI:** 10.1038/s41598-020-73867-7

**Published:** 2020-10-15

**Authors:** Christopher P. Morgan, Amol C. Shetty, Jennifer C. Chan, Dara S. Berger, Seth A. Ament, C. Neill Epperson, Tracy L. Bale

**Affiliations:** 1 Department of Pharmacology and Center for Epigenetic Research in Child Health and Brain Development, University of Maryland School of Medicine, Baltimore, MD 21201 USA; 2 Institute for Genome Sciences, University of Maryland School of Medicine, Baltimore, MD 21201 USA; 3 Department of Biomedical Sciences, Perelman School of Medicine, University of Pennsylvania, Philadelphia, PA 19104 USA; 4 Division of Reproductive Endocrinology and Infertility, Perelman School of Medicine, Department of Obstetrics and Gynecology, University of Pennsylvania, Philadelphia, PA 19104 USA; 5 Department of Psychiatry, University of Colorado School of Medicine, CU-Anschutz Medical Campus, Aurora, CO 80045 USA; 6 Departments of Pharmacology and Psychiatry, Center for Epigenetic Research in Child Health and Brain Development, HSF3, Room 9-171, University of Maryland School of Medicine, 670 W. Baltimore St., Baltimore, MD 21201 USA

**Keywords:** Translational research, Non-coding RNAs, Transcriptomics, Germline development, Developmental biology

## Abstract

Epidemiological studies from the last century have drawn strong associations between paternal life experiences and offspring health and disease outcomes. Recent studies have demonstrated sperm small non-coding RNA (sncRNA) populations vary in response to diverse paternal insults. However, for studies in retrospective or prospective human cohorts to identify changes in paternal germ cell epigenetics in association with offspring disease risk, a framework must first be built with insight into the expected biological variation inherent in human populations. In other words, how will we know what to look for if we don’t first know what is stable and what is dynamic, and what is consistent within and between men over time? From sperm samples from a ‘normative’ cohort of healthy human subjects collected repeatedly from each subject over 6 months, 17 healthy male participants met inclusion criteria and completed donations and psychological evaluations of perceived stress monthly. sncRNAs (including miRNA, piRNA, and tRNA) isolated from mature sperm from these samples were subjected to Illumina small RNA sequencing, aligned to subtype-specific reference transcriptomes, and quantified. The repeated measures design allowed us to define both within- and between-subject variation in the expression of 254 miRNA, 194 tRNA, and 937 piRNA in sperm over time. We developed screening criteria to identify a subset of potential environmentally responsive ‘dynamic’ sperm sncRNA. Implementing complex modeling of the relationships between individual dynamic sncRNA and perceived stress states in these data, we identified 5 miRNA (including let-7f-5p and miR-181a-5p) and 4 tRNA that are responsive to the dynamics of prior stress experience and fit our established mouse model. In the current study, we aligned repeated sampling of human sperm sncRNA expression data with concurrent measures of perceived stress as a novel framework that can now be applied across a range of studies focused on diverse environmental factors able to influence germ cell programming and potentially impact offspring development.

## Introduction

Epidemiological studies over the last several decades have provided strong evidence that parental life experiences shape offspring development, stimulating new consideration of the factors that underlie intergenerational programming of disease risk and resilience^1–16^. While these associations are well established, the molecular mechanisms involved remain elusive, especially for paternal transmission. Recent studies focusing on the influence of preconception paternal insults implicate epigenetic processes important for germ cell development and maturation^17–46^. Advances in high-throughput next-generation sequencing have revealed highly complex populations of RNA in sperm, and in animal studies, small noncoding RNA (sncRNA) populations have emerged as causal agents in the germline transmission of paternal experience^44,47–51^. Animal studies have provided the strongest evidence that paternal experiences, including various types of stress, drugs, and dietary manipulations, are associated with lasting changes in sperm RNA expression, where changes in specific miRNA and tRNA fragments have often been reported^17–25,27,28,31,32,36–38,40,41,52,53^. Further, injection of these RNAs into fertilized zygotes or incubation of sperm with RNA-containing extracellular vesicles, replicated the offspring phenotype, supporting a causal importance of sperm RNA changes^17,19–25^.

A growing number of human subject studies also demonstrated sperm sncRNA populations vary in association with diverse environmental exposures or experiences (e.g., smoking, diet, obesity, and stress/trauma)^28,39, 42,53–58^. However, the majority of these studies were constrained to a single timepoint or to a single within-subject comparison. Without time as a factor, it is challenging to determine environmentally-driven impact on sperm RNAs vs. population variance. Before we can begin searching for candidate sperm RNAs associated with or predictive of offspring disease risk (e.g., paternal stress or trauma and child autism or schizophrenia risk), a critical first step must be to develop a framework from a ‘normative’ dataset that includes within- and between-subject comparisons.

In the current studies, our goal was to assess the normative composition and dynamic changes in sperm sncRNA (including miRNA, piRNA, and tRNA) from a cohort of healthy human subjects from repeated monthly collections over 6 months. This repeated measures design allowed us to define both between-subject and within-subject variation in sperm sncRNA content with time as a factor. In addition, as our lab and others have previously demonstrated in animal models that sperm sncRNA are responsive to prior chronic stress experience, we modeled monthly transcriptomic data aligned with prior subject perceived stress state to identify specific sperm RNAs that fit strict criteria for consistent detection within- and between-subjects^17,18,21,28,31^. Unlike sperm DNA methylation, which is relatively stable over time within and between subjects, specific populations of sperm sncRNA appear to be more dynamic^59,60^. Our goal is that by providing the field with this comprehensive ‘normative’ dataset modeled with perceived stress, we can begin building a powerful framework to be utilized across cohorts and areas of study, and therefore novel and disease-predictive sncRNAs in human sperm will eventually be identified.

## Results

### Demographic and clinical characteristics of a normative cohort of males

**Figure 1:**
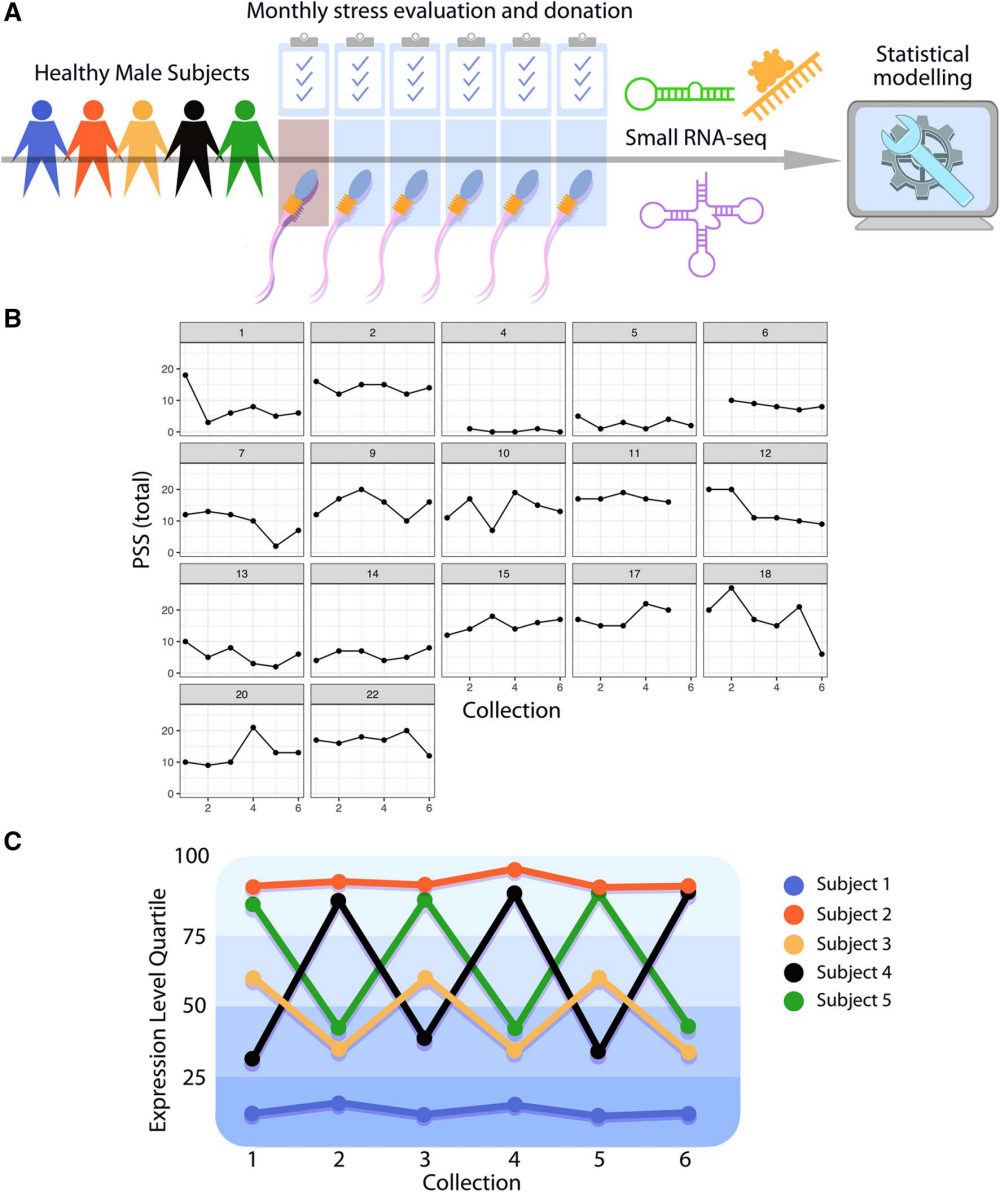
A normative cohort for within- and between-subject expression characteristics of sperm sncRNA populations associated with variable perceived stress experience across the 6-month study period. (**A**) To assess the normative composition and dynamic changes in human sperm small non-coding RNA (sncRNA) populations (including miRNA, tRNA, and piRNA), potential subjects were screened (indicated by brown shading of the first visit) to enroll healthy young men (ages 18–25) who completed psychological inventories, including the Perceived Stress Scale (PSS), and donated sperm monthly over 6 months (N = 17 subjects). Mature sperm was enriched from cryopreserved samples collected from these individuals, then sncRNA was isolated and subject to small RNA sequencing, followed by analysis and statistical modeling of sncRNA expression data and PSS scores. (**B**) On the same day of each monthly sperm collection, subjects also completed the Perceived Stress Scale (PSS) as part of their psychological inventory. The total score of this clinically validated measure of perceived stress score for each month (y-axis) is plotted over time (x-axis) for each subject (indicated by plot numbering). (**C**) Hypothetical expression plot of a sncRNA for 5 subjects over 6 months to illustrate the within- and between-subject structure and analysis of the data collected in our study. This structure was exploited to develop screening criteria for the identification of potential environmentally responsive ‘dynamic’ sperm sncRNA. These criteria were based on three a priori assumptions. First, we focused on sncRNA with a high degree of within-subject variation across time, assuming variation reflects responses to external stimuli. Second, we assumed that for a ‘dynamic’ sncRNA to be functionally relevant, it should also be expressed at relatively high levels (within the top quartile of expression) during at least one time point over the 6 months for a given subject. Third, we assumed sncRNA meeting these first two criteria (variable and relevant expression level) in multiple subjects within the final cohort were more likely to reflect a conserved response to variation in extrinsic factors in the environment, and therefore meet our final criteria as ‘dynamic’ to be tested in our statistical modeling. In the hypothetical plot provided, potential sncRNA expression patterns shown include: low expression and low variation in Subject 1, relevant high expression but low variation in Subject 2, low expression and high variation in Subject 3, relevant high expression and high variation in Subjects 4 and 5, exhibiting between-subject overlap. (**A**,**C)** by Tim Phelps © 2020 JHU AAM. (**B**) was generated using R (version 3.5.2) and the package ‘ggplot2’ (version 3.2.0) (https://cran.r-project.org/).

To establish the baseline characteristics of the sperm sncRNA complement, we recruited men from a relatively homogenous and ‘normative’ population of University of Pennsylvania students. Subjects between the ages of 18 and 25 were screened and excluded for major medical illness, mental health diagnoses, psychotropic medication use, and substance abuse. Following screening and baseline assessments, enrolled subjects returned monthly for 6 visits to donate semen samples for sperm sncRNA analysis. In addition, with each sample donation subjects completed psychological inventories, including the Perceived Stress Scale (PSS) (Fig. 1A,B). The PSS is commonly used to assess perception of stress over the previous month. It is the most commonly used psychological instrument to measure the degree to which situations in a subject’s life are appraised as stressful and taps into how unpredictable, uncontrollable and overloaded respondents experience their lives^61^. Baseline demographics and results from an Adverse Childhood Experiences (ACE) questionnaire and State-Trait Anxiety Inventory (STAI) demonstrate the final study cohort (N = 17) was relatively homogenous (Table 1). Subjects were between 19–25 years old (mean = 22.8, SD = 1.8), single, and without children. In addition, most subjects had ACE scores of 0, while only one subject had an ACE = 1 and two had an ACE = 2. Subjects scored between 22–39 on the STAI-Trait questionnaire (mean = 30.7, SD = 6.0). Mature sperm was enriched from cryopreserved samples collected from these individuals, then sncRNA was isolated and subject to small RNA sequencing. Sample characteristics are listed in Supplemental Table 1.

**Table 1:** Baseline demographics and assessments including ACE questionnaire and STAI inventory for study subjects.

Subject	Age	Marital status	Children	Race/ethnicity	Education	Adverse childhood events (ACE) (total)	State/Trait Anxiety Inventory (STAI) trait score
1	19	Single	No children	Asian	Some college	0	22
2	19	Single	No children	Asian	Some college	0	28
4	22	Single	No Children	Caucasian (non-Hispanic)	College degree	1	23
5	22	Single	No children	Caucasian (non-Hispanic)	Some college	0	23
6	23	Single	No children	Caucasian (non-Hispanic)	College degree	0	26
7	25	Single	No children	Hispanic	College degree	0	35
9	25	Single	No children	Caucasian (non-Hispanic)	Some graduate/professional	0	27
10	24	Single	No children	Caucasian (non-Hispanic)	Some graduate/professional	0	33
11	22	Single	No children	Caucasian (non-Hispanic)	College degree	0	38
12	24	Single	No children	Caucasian (non-Hispanic)	Some graduate/professional	0	31
13	25	Single	No children	Caucasian (non-Hispanic)	Some graduate/professional	0	27
14	22	Single	No children	Caucasian (non-Hispanic)	Some graduate/professional	0	24
15	24	Single	No children	Caucasian (non-Hispanic)	Some graduate/professional	0	37
17	23	Single	No children	Caucasian (non-Hispanic)	Some graduate/professional	2	34
18	23	Single	No children	Caucasian (non-Hispanic)	Some graduate/professional	2	38
20	23	Single	No children	Caucasian (non-Hispanic)	Some graduate/professional	0	39
22	22	Single	No children	Asian	Some graduate/professional	0	37

### Quality control and alignment to the Ensembl ncRNA transcriptome

Mature sperm cells are generally thought to be transcriptionally inert. As a result, the RNA content of mature sperm differs from most cells in several ways. The mature sperm RNA complement is dominated by sncRNA and lacks intact ribosomal RNA. This is likely due to the active degradation of specific pools of RNA or the protection of specific species from a broader degradation. These characteristics present a challenge for efforts to characterize sperm RNA populations, largely driven by a poor understanding of the functional role of sncRNA and a parallel dearth of annotations for sncRNA in standard reference genomes. In an effort to circumvent this lack in annotation, we have instead aligned our sequencing data to specific sncRNA transcriptomes curated to contain known or predicted sncRNA species. Initially, a dataset consisting of reads aligned to the Ensembl ncRNA reference transcriptome was used to establish various quality control criteria to apply across sperm sncRNA transcriptomes of interest (miRNA, piRNA, and tRNA). These included filtering criteria to exclude sncRNA features not consistently present across samples and multivariate analyses to identify samples that would be excluded as outliers for technical reasons. Sequencing libraries were generated from 100 samples collected from 17 subjects. In addition, libraries were generated from a common pool of sperm RNA for each round of library preparation and sequencing to serve as technical replicates to assess potential batch effects. These libraries were sequenced to an average depth of 8.2 × 10^6^ reads. Across samples, 29% of these reads aligned to 37,110 features in the Ensembl ncRNA reference transcriptome (Supplemental Table 1). To balance the detection of low-abundance transcripts against the characterization of transcripts consistently present across sperm samples, we retained transcripts with abundances ≥ 1 CPM in at least 75% of samples. This filtering criterion resulted in a near normal distribution of the log2 expression (log2 CPM) of 11,074 retained features (Supplemental Fig 1). Hierarchical clustering of samples based on ncRNA transcript expression, post-filtering and TMM normalization, identified samples 4-01 and 6-01 as outliers (Supplemental Fig 2A). This was likely driven by the large number of ncRNA features absent from these sample; in sample 4-01 and 6-01, 3695 and 969 features, respectively, had CPM = 0. The sample with the next largest number of absent features was 17-01 with 150; therefore 4-01 and 6-01 were excluded from further analyses. Though subjects were assigned to library prep/sequencing runs in a non-systematic manner, samples from the same subject were generally prepared and sequenced in the same run; therefore, it was important to test for batch effects. The clustering analysis grouped the 3 technical replicate samples into a single exclusive cluster, demonstrating any batch effects were minimal. The two outlier samples and the overlapping technical replicates were also identifiable in a Principal Component Analysis (PCA) when samples are plotted along the two components (PC1 and PC2) that account for the largest proportions of total variance in the dataset (37.3% and 12.2% respectively) (Supplemental Fig 2B).

**Figure 2:**
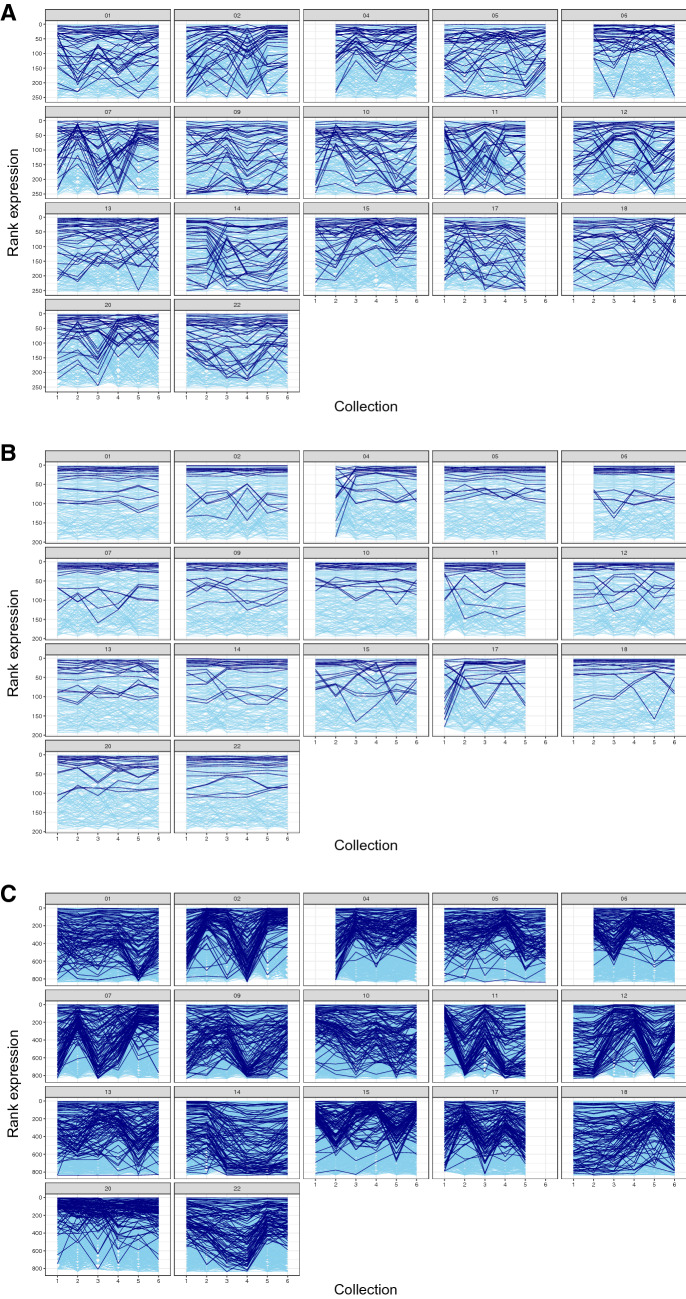
‘Dynamic’ sperm sncRNA populations display characteristic within-subject patterns of expression across time. Graphs represent the expression patterns of sperm sncRNA, the ranked expression (highest to lowest) of each (**A**) miRNA, (**B**) tRNA, and (**C**) piRNA is plotted by time within each subject. The final pool of ‘dynamic’ sncRNA (N = 33 miRNA, N = 17 tRNA, and N = 97 piRNA) are plotted in dark blue, while all other consistently expressed sncRNA are plotted in light blue. This figure generated using R (version 3.5.2) and the package ‘ggplot2’ (version 3.2.0) (https://cran.r-project.org/).

### Characterizing the class-specific sncRNA transcriptomes of sperm

There are 3 classes of sncRNA in sperm that are of particular relevance to the goals of the current study, miRNA, tRNA, and piRNA. None of these classes are present in their entirety within the Ensembl ncRNA transcriptome, therefore we aligned our sequencing data separately to more comprehensive class-specific reference transcriptomes. For miRNA, reads were initially aligned to 2657 features in the reference transcriptome obtained from miRbase. After filtering for features consistently present across samples (CPM ≥ 1 in 75% of samples), we identified 254 total miRNA in mature sperm. For tRNA, reads initially aligned to 425 features in the genomic tRNA database reference transcriptome from GtRNAdb. After filtering, we identified 194 total tRNA consistently present in mature sperm. For piRNA, reads initially aligned to 32,827 features in the reference transcriptome obtained from piRbase. After filtering, 837 total piRNA were consistently present in mature sperm. Hierarchical clustering and PCA demonstrate broad clustering of samples from the same subject, suggesting between-subject variation in sperm sncRNA expression is greater than within-subject variation (Supplemental Figs 3, 4, and 5). For miRNA, 70% of samples (N = 69) were grouped in clusters exclusively with samples from the same subject (Supplemental Fig 3A). These clusters ranged in size from 2 to 6 samples. The same was true for 78% of samples (N = 76) based on tRNA expression (Supplemental Fig 4A) and 66% of samples (N = 65) based on piRNA expression (Supplemental Fig 5A). This makes sense biologically, as between-subject variation is likely driven by the combination of differences in genetic background and past life experience, which should be greater than any changes in experience over the course of the 6 monthly collections.

**Figure 3:**
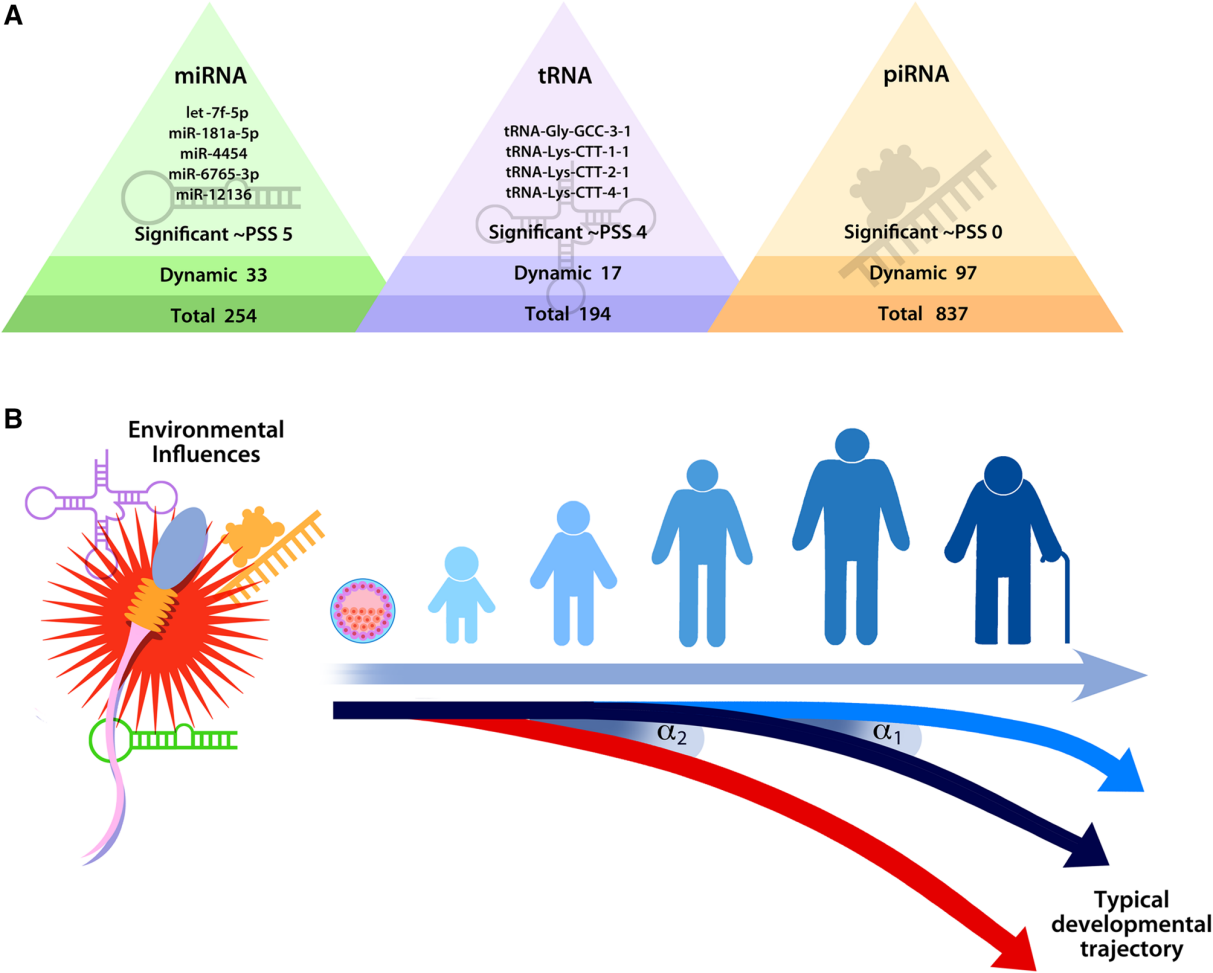
Individual ‘dynamic’ sperm miRNA and tRNA, but not piRNA, are significantly correlated with perceived stress experience, potentially acting to influence the earliest stages of development to shape long-term health outcomes. (**A**) Summary of analysis results, including the class-specific numbers of consistently expressed (total) and ‘dynamic’ sperm sncRNA, and the number of ‘dynamic’ sncRNA with statistically significant associations with perceived stress experience. (**B**) Theoretical model linking a chain of events beginning with an environmental experience or exposure, including stress, impacting sperm sncRNA levels. Acting individually or interacting together as part of a broader sncRNA code, these ‘dynamic’ sncRNA are able to transmit the encoded information regarding the paternal environment to offspring, potentially impacting developmental processes (e.g., rates of fertility, embryo division and implantation). Even if the initial impact of these ‘dynamic’ sperm sncRNA was small (indicated by ⍺1 or ⍺2), consequent shifts in the timing of developmental windows of susceptibility to additional events could produce significant differences over time, either in positive (resilience, indicated in blue) or negative (risk, indicated in red) directions when compared to a typical developmental trajectory (as indicated in black). Illustrations by Tim Phelps © 2020 JHU AAM.

To identify the top expressed features, each class of sperm sncRNA was ranked by expression from highest to lowest. The top 50 of each class are displayed in Tables 2, 3, and 4. To examine the stability of these rankings, we looked at how many months individual sncRNA were ranked in the top quartile of expression for each subject. Plotting a histogram of the number of months in which a given sncRNA was expressed in the top quartile of its class demonstrates that sncRNA expressed at a high level in at least 1 month were most likely to be highly expressed in all 6 collections from a subject (Supplemental Figs 6A, 6C, and 6E). The only subjects for which this was not the case were subject 11 for miRNA and piRNA and subject 17 for tRNA. The same is not true for sncRNA expressed at levels in the bottom quartile (Supplemental Figs 6B, 6D, and 6F).

**Table 2:** Top expressed miRNA.

Order	miRNA	Avg rank expression	Avg expression (CPM)	Avg rank CV expression (within-subject)	Avg CV expression (within-subject)
1	hsa-miR-10b-5p	2	616	132	0.45
2	hsa-miR-10a-5p	4	456	159	0.41
3	hsa-miR-22-3p	5	344	163	0.39
4	hsa-miR-12136	6	380	102	0.52
5	hsa-miR-30a-5p	13	144	160	0.41
6	hsa-miR-375-3p	13	193	99	0.52
7	hsa-miR-182-5p	14	133	159	0.41
8	hsa-miR-26a-5p	15	128	144	0.43
9	hsa-miR-10400-5p	15	120	205	0.3
10	hsa-miR-16-5p	16	122	167	0.39
11	hsa-miR-7977	17	259	120	0.51
12	hsa-miR-3960	19	93	195	0.31
13	hsa-miR-34c-5p	20	125	130	0.47
14	hsa-miR-148a-3p	21	128	102	0.51
15	hsa-miR-4800-3p	22	92	193	0.33
16	hsa-miR-1260a	23	173	121	0.51
17	hsa-miR-92a-3p	23	92	116	0.47
18	hsa-miR-542-3p	24	87	127	0.45
19	hsa-miR-25-3p	25	71	176	0.38
20	hsa-miR-27b-3p	26	78	98	0.58
21	hsa-miR-191-5p	27	67	152	0.42
22	hsa-miR-4634	27	58	220	0.24
23	hsa-miR-21-5p	27	83	91	0.55
24	hsa-miR-449a	31	75	100	0.54
25	hsa-miR-7704	32	54	200	0.32
26	hsa-miR-6765-3p	33	88	97	0.58
27	hsa-let-7a-5p	36	45	134	0.45
28	hsa-miR-141-3p	36	102	45	0.69
29	hsa-miR-1260b	38	68	121	0.49
30	hsa-miR-619-5p	38	36	178	0.34
31	hsa-miR-3614-3p	39	37	139	0.42
32	hsa-miR-30d-5p	43	35	164	0.4
33	hsa-miR-4787-5p	43	31	190	0.32
34	hsa-let-7f-5p	45	43	101	0.51
35	hsa-miR-28-3p	48	29	156	0.41
36	hsa-miR-2392	49	59	114	0.59
37	hsa-miR-3184-3p	49	39	84	0.53
38	hsa-miR-423-5p	49	39	84	0.53
39	hsa-miR-4763-5p	49	26	151	0.4
40	hsa-miR-192-5p	52	29	157	0.42
41	hsa-miR-151a-5p	54	24	164	0.39
42	hsa-miR-4771	56	22	173	0.36
43	hsa-miR-6511a-5p	56	24	138	0.43
44	hsa-miR-4516	56	21	196	0.26
45	hsa-miR-6836-3p	58	19	214	0.27
46	hsa-miR-6511b-5p	58	23	136	0.44
47	hsa-miR-149-3p	59	19	182	0.3
48	hsa-miR-186-5p	61	23	134	0.45
49	hsa-miR-10394-3p	62	18	206	0.29
50	hsa-miR-6790-3p	62	21	138	0.44

**Table 3:** Top expressed tRNA.

Order	tRNA	Avg rank expression	Avg expression (CPM)	Avg rank CV expression (within-subject)	Avg CV expression (within-subject)
1	hsa-tRNA-Glu-CTC-1-1	1	4994	92	0.43
2	hsa-tRNA-Glu-CTC-2-1	2	4936	90	0.43
3	hsa-tRNA-Glu-TTC-2-1	5	1940	124	0.34
4	hsa-tRNA-Lys-CTT-1-1	7	3231	49	0.64
5	hsa-tRNA-iMet-CAT-1-1	7	1276	113	0.38
6	hsa-tRNA-Gly-CCC-1-1	8	1082	64	0.51
7	hsa-tRNA-Lys-CTT-4-1	8	3148	47	0.65
8	hsa-tRNA-iMet-CAT-2-1	9	1169	105	0.40
9	hsa-tRNA-Gly-GCC-1-1	10	923	69	0.52
10	hsa-nmt-tRNA-Leu-TAA-4-1	11	720	67	0.47
11	hsa-nmt-tRNA-Leu-TAA-1-1	12	616	112	0.36
12	hsa-tRNA-SeC-TCA-1-1	13	501	155	0.22
13	hsa-tRNA-Gly-GCC-2-1	15	659	59	0.57
14	hsa-tRNA-Glu-TTC-1-1	17	303	150	0.25
15	hsa-tRNA-Gly-CCC-2-1	17	304	153	0.23
16	hsa-tRNA-Gly-GCC-3-1	19	567	48	0.63
17	hsa-tRNA-Gly-CCC-6-1	19	330	81	0.42
18	hsa-tRNA-Leu-CAG-2-1	20	220	135	0.29
19	hsa-tRNA-Leu-CAG-1-1	21	213	133	0.30
20	hsa-nmt-tRNA-Gln-TTG-6-1	21	401	79	0.47
21	hsa-tRNA-Asp-GTC-2-1	21	208	131	0.30
22	hsa-tRNA-His-GTG-1-1	23	177	106	0.39
23	hsa-tRNA-Thr-CGT-chr1-118	25	197	89	0.44
24	hsa-tRNA-Lys-CTT-2-1	25	322	60	0.54
25	hsa-tRNA-Arg-ACG-1-1	30	108	137	0.25
26	hsa-tRNA-Arg-ACG-2-1	30	110	150	0.22
27	hsa-tRNA-SeC-TCA-2-1	31	132	130	0.29
28	hsa-tRNA-Lys-TTT-3-1	34	95	131	0.30
29	hsa-tRNA-Arg-TCG-1-1	35	99	160	0.22
30	hsa-tRNA-Val-AAC-2-1	35	86	131	0.29
31	hsa-tRNA-Pro-TGG-3-1	37	120	95	0.45
32	hsa-tRNA-Leu-TAG-2-1	37	83	117	0.33
33	hsa-tRNA-Glu-TTC-4-1	38	76	157	0.22
34	hsa-tRNA-Lys-TTT-5-1	38	75	125	0.31
35	hsa-tRNA-Gln-TTG-1-1	38	65	159	0.22
36	hsa-tRNA-Lys-CTT-6-1	40	84	119	0.33
37	hsa-tRNA-Gln-CTG-1-1	41	70	110	0.35
38	hsa-tRNA-Gln-TTG-3-1	42	57	163	0.21
39	hsa-tRNA-Asp-GTC-3-1	43	54	125	0.33
40	hsa-tRNA-Arg-CCG-2-1	44	66	127	0.34
41	hsa-tRNA-Leu-TAG-3-1	46	51	140	0.28
42	hsa-nmt-tRNA-Gln-TTG-9-1	47	57	112	0.33
43	hsa-tRNA-Gln-CTG-2-1	47	63	99	0.39
44	hsa-tRNA-Leu-TAA-1-1	47	46	159	0.23
45	hsa-tRNA-Arg-CCT-4-1	49	47	146	0.27
46	hsa-tRNA-Pro-AGG-1-1	50	95	82	0.50
47	hsa-tRNA-Ser-GCT-3-1	51	54	147	0.25
48	hsa-tRNA-Val-TAC-1-1	51	40	151	0.25
49	hsa-tRNA-Gly-TCC-1-1	58	39	96	0.39
50	hsa-tRNA-Arg-CCG-1-1	60	38	112	0.36

**Table 4:** Top expressed piRNA.

Order	piRNA	Avg rank expression	Avg expression (CPM)	Avg rank CV expression (within-subject)	Avg CV expression (within-subject)
1	hsa-piR-24925	1	24,945	546	0.31
2	hsa-piR-3173	3	966	806	0.12
3	hsa-piR-27105	7	549	750	0.19
4	hsa-piR-23863	9	761	549	0.35
5	hsa-piR-22355	9	488	741	0.20
6	hsa-piR-165	13	383	696	0.23
7	hsa-piR-19619	13	394	627	0.28
8	hsa-piR-24713	15	326	734	0.19
9	hsa-piR-20684	15	308	728	0.19
10	hsa-piR-7034	15	507	385	0.43
11	hsa-piR-9359	16	506	384	0.43
12	hsa-piR-6266	18	287	645	0.25
13	hsa-piR-12106	20	258	699	0.22
14	hsa-piR-10365	20	227	741	0.17
15	hsa-piR-26211	20	767	295	0.51
16	hsa-piR-2465	22	221	653	0.26
17	hsa-piR-9471	25	233	555	0.33
18	hsa-piR-28905	26	205	546	0.36
19	hsa-piR-3351	27	188	670	0.25
20	hsa-piR-12078	27	225	357	0.46
21	hsa-piR-31148	28	369	140	0.76
22	hsa-piR-25836	29	153	656	0.25
23	hsa-piR-14727	29	224	581	0.30
24	hsa-piR-3953	30	196	399	0.42
25	hsa-piR-28241	34	150	615	0.28
26	hsa-piR-25596	35	173	414	0.43
27	hsa-piR-8336	38	149	393	0.45
28	hsa-piR-22784	40	175	185	0.62
29	hsa-piR-23494	40	361	391	0.45
30	hsa-piR-2251	42	100	734	0.20
31	hsa-piR-15477	42	286	375	0.49
32	hsa-piR-22404	43	104	647	0.26
33	hsa-piR-6428	43	236	366	0.64
34	hsa-piR-22183	44	107	633	0.27
35	hsa-piR-20422	45	92	694	0.22
36	hsa-piR-10341	49	146	451	0.42
37	hsa-piR-10996	50	95	484	0.37
38	hsa-piR-8052	50	76	719	0.21
39	hsa-piR-263	51	87	613	0.29
40	hsa-piR-15207	54	72	773	0.17
41	hsa-piR-22921	57	111	472	0.38
42	hsa-piR-8736	58	73	690	0.24
43	hsa-piR-11637	63	84	523	0.39
44	hsa-piR-30999	70	88	267	0.54
45	hsa-piR-2955	76	54	624	0.27
46	hsa-piR-1988	77	76	507	0.41
47	hsa-piR-5570	77	85	242	0.56
48	hsa-piR-26090	77	87	400	0.46
49	hsa-piR-9327	78	50	727	0.19
50	hsa-piR-2073	79	64	555	0.33

### ‘Dynamic’ sperm sncRNA

To identify potential environmentally responsive ‘dynamic’ sncRNA, we developed screening criteria based on three a priori assumptions: (1) we assumed ‘dynamic’ sncRNA were likely to exhibit a higher degree of variation in expression over time in response to changes in the environment; (2) given the disparity in the amount of RNA present in sperm relative to ovum, we assumed sncRNA with the potential to impact offspring development would need to be highly expressed; and (3) we assumed sncRNA meeting criteria based on these first two assumptions in multiple subjects were more likely to reflect a conserved functional response to extrinsic factors^62–64^. The analysis we performed to characterize these properties and identify ‘dynamic’ sncRNA is illustrated in Fig. 1C and described in greater detail in the “Methods”. An initial pool of candidates consisted of sncRNA that exhibited within-subject variation (CV expression) ranked in the top quartile of each class of features, while also being expressed in the top quartile in at least one timepoint over the 6 months for a given subject. One hundred seventeen miRNA, 75 tRNA, and 369 piRNA met these criteria in at least one subject. The expression of these initial candidates is plotted for each subject in Supplemental Fig 7. We then asked how many of these initial candidates overlapped between subjects (Supplemental Fig 8). Thirty-three miRNA, 17 tRNA, and 97 piRNA met the within-subject criteria in at least 25% (N = 5) of subjects (count candidate ≥ 5), constituting our final pool of ‘dynamic’ sperm sncRNA. These sncRNA are displayed in Tables 5, 6, and 7 and their expression over time is plotted for each subject in Fig. 2A–C, respectively.

**Table 5:** 'Dynamic' sperm miRNA.

Order	miRNA	Avg rank expression	Avg expression (CPM)	Avg rank CV expression (within-subject)	Avg CV expression (within-subject)	Count candidate
1	hsa-miR-12136	6	380	102	0.52	5
2	hsa-miR-375-3p	13	193	99	0.52	5
3	hsa-miR-148a-3p	21	128	102	0.51	7
4	hsa-miR-27b-3p	26	78	98	0.58	7
5	hsa-miR-21-5p	27	83	91	0.55	7
6	hsa-miR-449a	31	75	100	0.54	6
7	hsa-miR-6765-3p	33	88	97	0.58	6
8	hsa-miR-141-3p	36	102	45	0.69	12
9	hsa-let-7f-5p	45	43	101	0.51	5
10	hsa-miR-3184-3p	49	39	84	0.53	7
11	hsa-miR-423-5p	49	39	84	0.53	7
12	hsa-miR-3907	65	32	82	0.57	8
13	hsa-miR-1208	65	24	110	0.54	5
14	hsa-miR-4454	69	27	91	0.54	5
15	hsa-miR-891a-5p	76	31	73	0.70	8
16	hsa-miR-363-3p	80	29	57	0.63	8
17	hsa-miR-3182	81	17	94	0.54	8
18	hsa-miR-6090	84	22	64	0.72	9
19	hsa-miR-6750-3p	88	19	54	0.67	7
20	hsa-miR-203a-3p	103	30	31	0.98	11
21	hsa-miR-181a-5p	106	33	75	0.63	7
22	hsa-miR-203b-5p	107	30	29	0.99	10
23	hsa-miR-130a-3p	107	11	119	0.52	5
24	hsa-let-7g-5p	108	11	102	0.52	5
25	hsa-miR-320a-3p	111	17	57	0.75	8
26	hsa-miR-1247-3p	116	11	60	0.66	8
27	hsa-miR-320b	129	14	43	0.80	9
28	hsa-miR-6880-5p	133	8	73	0.61	7
29	hsa-miR-23a-3p	142	11	57	0.81	6
30	hsa-miR-23b-3p	144	11	58	0.79	6
31	hsa-miR-29a-3p	151	8	65	0.62	5
32	hsa-miR-4757-5p	151	9	42	0.84	6
33	hsa-miR-6731-5p	160	7	53	0.87	7

**Table 6:** 'Dynamic' sperm tRNA.

Order	tRNA	Avg rank expression	Avg expression (CPM)	Avg rank CV expression (within-subject)	Avg CV expression (within-subject)	Count candidate
1	hsa-tRNA-Lys-CTT-1-1	7	3231	49	0.64	10
2	hsa-tRNA-Gly-CCC-1-1	8	1082	64	0.51	6
3	hsa-tRNA-Lys-CTT-4-1	8	3148	47	0.65	10
4	hsa-tRNA-Gly-GCC-1-1	10	923	69	0.52	6
5	hsa-nmt-tRNA-Leu-TAA-4-1	11	720	67	0.47	10
6	hsa-tRNA-Gly-GCC-2-1	15	659	59	0.57	9
7	hsa-tRNA-Gly-GCC-3-1	19	567	48	0.63	10
8	hsa-tRNA-Gly-CCC-6-1	19	330	81	0.42	7
9	hsa-nmt-tRNA-Gln-TTG-6-1	21	401	79	0.47	6
10	hsa-tRNA-Lys-CTT-2-1	25	322	60	0.54	6
11	hsa-tRNA-Pro-TGG-3-1	37	120	95	0.45	5
12	hsa-tRNA-Pro-AGG-1-1	50	95	82	0.50	6
13	hsa-tRNA-Asn-GTT-15-1	69	28	95	0.41	5
14	hsa-tRNA-Asn-GTT-19-1	70	28	94	0.42	5
15	hsa-nmt-tRNA-Ser-TGA-3-1	71	31	48	0.61	6
16	hsa-tRNA-Und-NNN-4-1	80	20	51	0.61	5
17	hsa-tRNA-Glu-TTC-10-1	89	17	37	0.68	5

**Table 7:** 'Dynamic' sperm piRNA.

Order	piRNA	Avg rank expression	Avg expression (CPM)	Avg rank CV expression (within-subject)	Avg CV expression (within-subject)	Count candidate
1	hsa-piR-26211	20	767	295	0.51	6
2	hsa-piR-31148	28	369	140	0.76	14
3	hsa-piR-22784	40	175	185	0.62	10
4	hsa-piR-15477	42	286	375	0.49	5
5	hsa-piR-6428	43	236	366	0.64	7
6	hsa-piR-30999	70	88	267	0.54	8
7	hsa-piR-5570	77	85	242	0.56	9
8	hsa-piR-5304	79	121	318	0.65	7
9	hsa-piR-4648	101	69	338	0.52	5
10	hsa-piR-17025	101	149	313	0.70	8
11	hsa-piR-8891	114	117	372	0.67	7
12	hsa-piR-18089	114	83	244	0.69	9
13	hsa-piR-772	119	60	384	0.56	6
14	hsa-piR-28626	120	61	169	0.69	14
15	hsa-piR-14072	126	74	141	0.77	13
16	hsa-piR-26349	146	35	370	0.49	6
17	hsa-piR-30538	154	36	254	0.54	8
18	hsa-piR-875	175	30	329	0.53	6
19	hsa-piR-19697	187	25	302	0.50	6
20	hsa-piR-23716	189	37	125	0.79	16
21	hsa-piR-6191	189	37	243	0.59	11
22	hsa-piR-28044	199	57	71	0.93	14
23	hsa-piR-19009	203	37	127	0.78	14
24	hsa-piR-23367	210	21	431	0.47	5
25	hsa-piR-10037	213	27	167	0.72	14
26	hsa-piR-4898	217	25	457	0.48	5
27	hsa-piR-31194	218	25	455	0.49	5
28	hsa-piR-18612	220	19	438	0.42	5
29	hsa-piR-27695	227	22	404	0.44	5
30	hsa-piR-1129	235	45	281	0.63	8
31	hsa-piR-20280	252	21	267	0.55	6
32	hsa-piR-1386	260	17	268	0.57	6
33	hsa-piR-445	268	16	278	0.56	6
34	hsa-piR-20373	269	14	350	0.48	6
35	hsa-piR-16119	270	59	90	0.82	12
36	hsa-piR-18998	270	59	90	0.82	12
37	hsa-piR-13390	271	14	364	0.49	7
38	hsa-piR-608	272	22	100	0.82	14
39	hsa-piR-27282	274	25	112	0.82	11
40	hsa-piR-17850	278	24	116	0.82	13
41	hsa-piR-6527	279	20	142	0.75	12
42	hsa-piR-1823	288	27	298	0.65	8
43	hsa-piR-15	290	25	93	0.86	13
44	hsa-piR-4673	292	17	367	0.54	5
45	hsa-piR-13152	306	16	218	0.61	6
46	hsa-piR-19503	308	16	353	0.55	5
47	hsa-piR-14706	312	11	378	0.46	6
48	hsa-piR-13606	325	13	340	0.49	5
49	hsa-piR-6999	326	18	110	0.87	11
50	hsa-piR-21337	328	36	246	0.71	8
51	hsa-piR-20083	336	16	103	0.84	11
52	hsa-piR-3188	339	14	391	0.57	5
53	hsa-piR-5647	342	23	128	0.83	8
54	hsa-piR-24896	343	13	189	0.67	11
55	hsa-piR-22687	345	10	311	0.57	7
56	hsa-piR-8913	350	17	84	0.88	11
57	hsa-piR-15758	351	14	140	0.77	9
58	hsa-piR-10155	354	13	329	0.53	6
59	hsa-piR-29889	359	13	141	0.76	10
60	hsa-piR-1325	362	15	93	0.84	12
61	hsa-piR-20495	370	13	111	0.83	12
62	hsa-piR-29380	373	9	369	0.50	5
63	hsa-piR-19468	374	11	181	0.69	7
64	hsa-piR-13532	374	13	149	0.74	8
65	hsa-piR-30832	378	17	235	0.65	8
66	hsa-piR-1711	379	11	116	0.79	10
67	hsa-piR-6674	380	9	287	0.59	7
68	hsa-piR-15190	381	11	114	0.79	10
69	hsa-piR-30472	387	13	125	0.78	7
70	hsa-piR-7841	394	14	110	0.84	6
71	hsa-piR-4093	396	12	132	0.81	8
72	hsa-piR-21538	400	10	188	0.67	6
73	hsa-piR-4580	404	9	352	0.53	5
74	hsa-piR-23714	411	9	203	0.67	5
75	hsa-piR-21903	417	10	109	0.81	9
76	hsa-piR-28592	418	15	263	0.67	8
77	hsa-piR-12195	421	13	79	0.89	10
78	hsa-piR-20449	425	11	99	0.84	8
79	hsa-piR-12469	431	10	131	0.82	7
80	hsa-piR-28350	435	9	112	0.80	8
81	hsa-piR-7054	437	10	145	0.77	6
82	hsa-piR-9125	439	13	69	0.96	10
83	hsa-piR-18443	442	9	100	0.86	10
84	hsa-piR-16725	456	9	118	0.81	7
85	hsa-piR-3398	458	10	91	0.87	8
86	hsa-piR-28047	461	9	207	0.65	6
87	hsa-piR-12771	464	8	109	0.82	7
88	hsa-piR-26714	468	9	146	0.76	7
89	hsa-piR-24084	468	9	100	0.83	6
90	hsa-piR-2519	468	8	135	0.81	7
91	hsa-piR-10779	473	7	225	0.62	5
92	hsa-piR-19755	475	7	231	0.61	5
93	hsa-piR-28297	496	7	143	0.79	5
94	hsa-piR-20709	496	7	159	0.72	5
95	hsa-piR-27113	512	8	137	0.77	6
96	hsa-piR-19035	519	8	76	0.94	6
97	hsa-piR-8490	550	7	242	0.69	6

### Perceived stress and ‘dynamic’ sperm sncRNA

To assess the potential relationship between perceived stress and individual ‘dynamic’ sncRNA, we conducted a series of linear models to test for relationships between ‘dynamic’ sncRNA expression and PSS scores. Based on data from our lab in a mouse model of paternal stress, we hypothesized that there would be a delay in the impact of stress experience on the expression of ‘dynamic’ sncRNA^17^. Therefore, we evaluated the following seven relationships for an individual sncRNA’s expression level in a sperm sample and: (1) PSS score at the time it was collected (t), (2) PSS score at the time of the previous collection (t − 1), (3) PSS score at t − 2, (4) PSS score at t − 3, (5) change in PSS score between t and t − 1, (6) change in PSS score between t and t − 2, and (7) change in PSS score between t and t − 3. The interpretation of the first four models was that the level of a sncRNA changes in a direct relationship with PSS score, possibly with a delay (i.e., a rheostat model), whereas the last three model an increase or decrease in a sncRNA relative to the magnitude of a change in PSS score over the specified period (i.e., a change detector). Ultimately, we identified five ‘dynamic’ miRNA with associations to PSS scores that passed our significance cutoffs (p < 0.01 and FDR < 0.2): let-7f-5p, miR-181a-5p, miR-4454, miR-6765-3p, and miR-12136 (Table 8). We identified four ‘dynamic’ tRNA with significant associations to PSS scores: tRNA-Gly-GCC-3-1, tRNA-Lys-CTT-1-1, tRNA-Lys-CTT-2-1, and tRNA-Lys-CTT-4-1 (Table 8). There were no significant associations between ‘dynamic’ piRNA and PSS scores (Fig. 3A).

**Table 8:** Characteristics of linear fixed effects models assessing the relationship between perceived stress experience and the expression of ‘dynamic’ sncRNA: Table includes model regression coefficients (log2FC), adjusted p-values, and FDR. Significant relationships are highlighted in bold.

Dynamic sncRNA	sncRNA ∼ PSS time of collection (t)			sncRNA ∼ PSS at t-1			sncRNA ∼ PSS at t-2			sncRNA ∼ PSS at t-3			sncRNA ∼ delta PSS t-1			sncRNA ∼ delta PSS t-2			sncRNA ∼ delta PSS t-3		
	log2FC	p (adjusted)	FDR	log2FC	p (adjusted)	FDR	log2FC	p (adjusted)	FDR	log2FC	p (adjusted)	FDR	log2FC	p (adjusted)	FDR	log2FC	p (adjusted)	FDR	log2FC	p (adjusted)	FDR
hsa-let-7f-5p	− **0.060**	**0.003**	**0.045**	− 0.053	0.013	0.220	− 0.041	0.104	0.343	− 0.038	0.211	0.700	− 0.009	0.754	0.998	− 0.008	0.104	0.839	− 0.034	0.394	0.744
hsa-miR-181a-5p	− **0.116**	**0.000**	**0.001**	− **0.103**	**0.001**	**0.019**	− **0.090**	**0.008**	**0.128**	− **0.113**	**0.005**	**0.078**	− 0.008	0.852	0.998	− 0.011	0.008	0.839	− 0.010	0.852	0.937
hsa-miR-4454	− 0.041	0.124	0.511	− 0.054	0.060	0.392	− **0.086**	**0.008**	**0.128**	− 0.091	0.017	0.115	− 0.002	0.958	0.998	0.060	0.008	0.683	0.071	0.157	0.633
hsa-miR-6765-3p	− 0.021	0.445	0.698	− 0.029	0.322	0.665	− 0.073	0.023	0.194	− **0.098**	**0.007**	**0.078**	− 0.015	0.700	0.998	0.037	0.023	0.683	0.085	0.079	0.633
hsa-miR-12136	− 0.033	0.072	0.338	− 0.040	0.045	0.392	− 0.052	0.028	0.194	− **0.080**	**0.004**	**0.078**	0.009	0.727	0.998	0.014	0.028	0.773	0.053	0.145	0.633
hsa-tRNA-Gly-GCC-3-1	0.022	0.370	0.897	0.044	0.056	0.342	0.067	0.011	0.075	0.077	0.012	0.087	− 0.024	0.421	0.854	− 0.083	0.013	0.054	− **0.104**	**0.005**	**0.023**
hsa-tRNA-Lys-CTT-1-1	0.007	0.838	0.950	0.034	0.202	0.382	0.064	0.031	0.087	0.077	0.031	0.087	− 0.036	0.329	0.854	− **0.104**	**0.006**	**0.037**	− **0.137**	**0.002**	**0.009**
hsa-tRNA-Lys-CTT-2-1	0.027	0.309	0.897	0.041	0.077	0.342	0.059	0.022	0.075	**0.083**	**0.007**	**0.087**	− 0.039	0.193	0.854	− **0.090**	**0.005**	**0.037**	− **0.130**	**0.001**	**0.009**
hsa-tRNA-Lys-CTT-4-1	0.011	0.746	0.906	0.039	0.162	0.382	0.071	0.019	0.075	0.083	0.019	0.087	− 0.034	0.365	0.854	− **0.107**	**0.006**	**0.037**	− **0.139**	**0.001**	**0.009**

Table includes model regression coefficients (log2FC), adjusted p-values, and FDR. Significant relationships (p < 0.01 and FDR < 0.2) are highlighted in bold.

## Discussion

There is a growing appreciation for the importance of the paternal preconception environment in the developmental programming of offspring^1,15,44,48–51^. Though the mechanisms underlying this developmental plasticity are likely adaptive, in the context of human health, these effects may be expressed as changes in disease risk or resilience. Recent work from our lab and others demonstrating direct causal associations between sperm RNA and complex offspring phenotypes have shifted the focus of the investigations into the mechanisms underlying intergenerational transmissions to sperm RNA^17,19–25^.

In the early 2000s, Stephen Krawetz and colleagues demonstrated that human sperm contained specific populations of RNA, that the RNA species present were conserved across healthy subjects, and that these RNA were delivered to the ovum, where they played a functional role in early zygote development^65,66^. Though the initial focus of this work was on protein-coding RNA, these studies also identified sncRNA in human sperm, including miRNA, tRNA, and piRNA, and as our understanding of the functional relevance of sncRNA in cell physiology has advanced, so too has our appreciation for the importance of sncRNA in the function of the germ cell and in regulating the earliest processes of newly fertilized zygotes^26,47,62,66–70^. A portion of the sncRNA present in sperm may only be remnants of spermatogenic processes, such as the RNA fragmentation products of ribosomal RNA subunits extensively degraded to suppress spurious protein translation in these transcriptionally quiescent cells^71^. However, it is clear that much of the sncRNA content of sperm is not the product of stochastic processes, but is actively shaped, in part, through interactions with extracellular vesicles (EVs) secreted by somatic cells along the reproductive tract, including the epididymis^63,72–74^.

Mechanistic studies in animal models show an association of changes in germ cell sncRNA with intergenerational transmission^17–25,27,28,31,32,36–38,40,41,52,53^. Several labs independently demonstrated that injecting total RNA isolated from sperm exposed to environmental manipulations, specific classes of sncRNA (often differentiated by size), or even specific environmentally-responsive sncRNA into newly fertilized zygotes was sufficient to transmit/phenocopy complex phenotypes in affected offspring^17,19–25^. Epidemiological studies suggest that similar processes may link paternal adverse experiences and offspring disease risk, but causal or prospective data are lacking^28,39,42,53–56^. Progress in the field has been held back by a lack of critical details regarding many of the necessary factors to design prospective clinical studies and test such hypotheses. There is a primary need to first understand fundamental dynamics of sperm transcriptomics. For example, to differentiate between variation driven by genetics vs. environment (intrinsic vs extrinsic factors), it is necessary to examine sperm content over multiple time points (within- vs between-subject comparisons). Therefore, we have established an extensive dataset describing the dynamics of sperm sncRNA expression over time and across a normative cohort of human subjects. Of course, defining any cohort as ‘normative’ can be problematic. Our selection criteria were developed to recruit a healthy group of males with minimal heterogenous and confounding characteristics. However, age can influence sperm epigenetics, including sncRNA content, and our cohort may not comprise the typical age distribution of males with reproductive intent^17,75–78^. In addition, the lived experience of people from different racial or ethnic groups can vary dramatically and are likely to influence the dynamics of specific sperm sncRNA expression. Future studies will need to determine how generalizable our current model is from this initial cohort across the diversity present in the broader population.

As has been previously reported, miRNA comprised a smaller fraction of the total sperm sncRNA pool in our subjects^54,56^. However, each miRNA can regulate the expression of hundreds of genes, and multiple miRNAs can collaborate in targeting extensive cellular processes and molecular pathways^79^. For example, we previously demonstrated that injecting a specific pool of 9 stress-sensitive miRNA into newly fertilized mouse zygotes extensively altered the expression of specific target stored maternal mRNA transcripts within 24 h^19^. In our current study, an average of 0.73% of total reads aligned to mature miRNA (compared to 29% aligning to the Ensembl ncRNA reference transcriptome), and after filtering, we identified a pool of 254 consistently expressed miRNA. We noted that several of the top-expressed miRNA in this pool have known functions in spermatogenesis or the epididymal maturation of sperm, including miR-10a-5p (consistently one of the highest expressed sperm miRNA), miR-30a-5p, and miR-26a-5p (84–86)^80–82^. Others may play important roles in the earliest stages of zygotic development^81^. For example, miR34c-5p is among the highest expressing sperm miRNA in humans and mice, and is required for the first cleavage division in mouse zygotes^68,83,84^. These studies suggest functional roles for sperm miRNA in biological processes important to reproduction and that may impact post-fertilization embryo development.

Our experimental design did not include manipulations of human subjects or interventions; instead, it was intended to build an initial framework from a ‘normative’ human subject cohort, including examination within subjects over time and comparisons between subjects. By collecting samples across an extended period, we anticipated exploiting variation in the experiences of participants to screen for environmentally responsive ‘dynamic’ sperm sncRNA. Using screening criteria based on three a priori assumptions, as detailed in the “Methods”, we identified 33 final ‘dynamic’ miRNA—highly expressed and with a sufficient dynamic pattern over time, both within- and between-subjects. In validation of our assumptions and selection criteria, we found that several of the ‘dynamic’ miRNA identified were also previously reported in sperm from rodents and humans, and associated with male environmental perturbations, including chronic stress^18,21,28^. For example, miR-449a levels were reduced in sperm from adult men who had experienced a high number of adverse childhood events and in the sperm of male mice following chronic stress^28^. Further, in two independent models, miR-375-3p was elevated in the sperm from adult male mice following prior chronic stress experience that occurred in the postnatal or pubertal/adult period^18,21^.

tRNA are an abundant class of sncRNA also found in mature sperm^73,84,85^. In our study, just over 2% of total reads aligned to tRNA, and we identified 194 consistently expressed tRNA. In addition to the role intact tRNA play in protein translation, tRNA-derived RNA fragments (tRFs) regulate gene expression, and in some instances act in concert with cellular machinery already in place for miRNA and piRNA actions^86–88^. In 2016, two studies implicated sperm tRFs in the epigenetic germline inheritance of complex metabolic phenotypes following paternal high fat or low protein dietary exposures in mice^23,25^. At ~ 75 bp, intact tRNA are significantly longer than either miRNA or piRNA, and also longer than the 36 bp read lengths of our sequencing dataset, and therefore, we were limited in our ability to differentiate between sequence reads derived from intact tRNA and those from tRFs^88^.

In examining the expression pattern of sperm tRNAs across subjects, it was clear that this class of sperm sncRNA is far less variable overall than either miRNA or piRNA. However, we did identify a subset of 17 sperm tRNA that met our criteria for ‘dynamic’ sncRNA. Focusing on the expression of these ‘dynamic’ tRNA over time, there were clearly subjects who had more variable expression than others, which might correlate with between-subject variation in environmental factors that were not examined in our study. It was also clear that the most ‘dynamic’ tRNA for a given subject tended to be present at lower overall expression levels. Three of the final ‘dynamic’ tRNA we identified, tRNA-Gly-GCC-1-1, tRNA-Gly-GCC-2-1, and tRNA-Gly-GCC-3-1, transport the same amino acid (glycine) and share the same anticodon sequence (i.e., isodecoders). Reduced sperm expression of tRFs derived from two other tRNA-Gly-GCC isodecoders were previously associated with poor embryo quality when those human sperm samples were used for in vitro fertilization, supporting an important biological function for these fragments in sperm^85^.

Consistent with previous reports, piRNA were the most prevalent class of sperm sncRNA in our dataset in terms of expression abundance^54,80^. An average of 4.9% of total sequence reads aligned to piRNA across subjects. In addition to comprising the largest proportion of the total sperm sncRNA pool, far more piRNA (837) were consistently expressed across sperm samples. This was not unexpected, as tens of thousands of human piRNA have been annotated^89^. piRNA are predominately expressed in the germline where they fulfill their canonical role in maintaining genomic stability by repressing repetitive elements; though there is a growing appreciation for the role piRNA play in the post-transcriptional regulation of protein-coding transcripts^80,90,91^. Interestingly, in *D. melanogaster* and *C. elegans*, piRNA play a key role in transgenerational epigenetic inheritance of specific traits^92,93^. In rodents, changes in sperm piRNA expression were reported in association with male dietary manipulations and early life chronic stress experiences^20,21,41^.

Of consistently expressed sperm piRNA, 97 met selection criteria and were categorized as ‘dynamic’. Compared to miRNA, little is known about the functional role of individual piRNA, especially in mature sperm. Interestingly, in our study, we noted that when viewed across time, piRNA expression patterns in many subjects appear to display a bi-monthly cycle. Similar but less apparent patterns were also noted in the miRNA plots. Supporting a potential biological relevance, 15 of the 97 ‘dynamic’ piRNA had a cyclical expression pattern in at least 25% of our subjects. Such an expression pattern may reflect cyclical changes in extrinsic environmental factors or an intrinsic rhythm in male fertility not previously described.

Recent studies from our lab and others have demonstrated that the sperm sncRNA content is responsive to prior stress experience^17,18,21,28,31^. Further, our recent molecular studies in mice identified the specific timing at which previously stressed males were able to transmit a specific phenotype to their offspring^17^. These studies demonstrated a key finding in the field, that intergenerational transmission may occur, and in some contexts may only occur after an extended period following the cessation of the insult. This was a critical piece of the puzzle for formulating hypotheses in modeling human subject data—when to expect a detectable change to show up in the sperm after a given experience? Therefore, we evaluated a series of statistical models to align our sncRNA expression data with that of a clinical measure of perceived stress. These analyses identified significant associations between the expression of several ‘dynamic’ sncRNA and either the subject’s absolute PSS score, concurrently or at previous timepoints (rheostatic model), or the change in PSS score between timepoints (delta-detector). Five ‘dynamic’ miRNA and four tRNA were determined to be significantly associated with perceived stress experience. Of these, we noted that several had been identified in previous studies as important, including the miRNA, let-7f-5p and miR-181a-5p, and the tRNA, tRNA-Gly-GCC-3-1, tRNA-Lys-CTT-1-1, tRNA-Lys-CTT-2-1, and tRNA-Lys-CTT-4-1^25,56,94^. The miRNA let-7f-5p and tRFs derived from tRNA-Gly-GCC and tRNA-Lys-CTT isodecoders were previously identified as differentially expressed in the sperm of male mice following exposure to a low-protein diet^25^. In that study, injection of a tRF derived from the 5′ end of tRNA-Gly-GCC depressed the levels of genes highly expressed in preimplantation embryos targeted by the endogenous retroelement MERVL. tRNA-Lys-CTT levels were increased in human sperm following a high sugar diet exposure^56^.

Of great relevance to our results, a recent study identified significant correlations between levels of prior childhood trauma and adult plasma miR-181a-5p levels and several members of the let-7 family, similar to our results in sperm^94^. This suggests that these miRNA may be part of an evolutionarily conserved stress-responsive mechanism, conserved across tissues and species. How this would impact post-conception reproduction or embryonic development is unknown, but one of the top predicted gene targets of these miRNA, protogenin (PRTG), could play a role^95–97^. PRTG is involved in regulating early embryonic developmental transitions and trophoblast differentiation, and has been associated with ADHD and measures of cognitive development in multiple studies^95,96,98,99^. Therefore, changes in levels of sperm let-7f-5p and miR-181a-5p delivered at fertilization could regulate PRTG expression, among other genes, impacting rates of embryo division and implantation. Even if these effects were small, consequent shifts in the timing of developmental windows of susceptibility to environmental signals could produce significant differences in development and health outcomes mapped onto genetic risk (as shown in the schematic in Fig. 3B).

## Conclusion

In these studies, we have utilized between- and within-subject sperm sncRNA comparisons and provided an initial framework onto which additional human subject data can be built. These data confirm high expressing common miRNA, tRNA, and piRNA that were dynamic in their pattern of expression over time, likely responsive to a factor in the internal or external environment. Further, using our perceived stress state analyses, we were able to identify miRNA and tRNA that fit strict modeling criteria for changing their expression levels following a previous perceived high stress state. Much work remains to be done in this field, but these data provide a powerful starting point. From here, it is conceivable that one day we may be able to map onto such a normative data set the sncRNA expression of fathers of children with neurodevelopmental disorders or men with traumatic experiences, such as returning from military service, and be able to identify biomarkers predictive of offspring developmental risk and resilience factors.

## Methods and materials

### Subject recruitment

A cohort of 18 healthy males was recruited from the University of Pennsylvania student body to establish normative sperm molecular signatures as a benchmark for comparison to later clinical populations. The study was approved by the Perelman School of Medicine at the University of Pennsylvania Institutional Review Board, all participants provided written informed consent, and all research was performed in accordance with relevant guidelines. Subjects between the ages of 18 and 25 were screened for history of major medical illnesses, mental health diagnoses, and substance abuse. Grounds for exclusion included: (1) history (participant self-report) of major medical illnesses or other current medical conditions that the physician investigator deemed as contraindicated for study participation; (2) regular or recreational use of any psychotropic medication (e.g., antidepressants, antipsychotics, psychostimulants or anxiolytics), as per self-report, (3) recent (within previous year) diagnosis (per MINI International Neuropsychiatric Interview) or treatment for any psychiatric disorder or substance use disorder (previous 2 years), (4) lifetime history of schizophrenia or other psychotic disorder, substance addiction disorder (excepting nicotine), (5) current use of any tobacco products, determined by urine cotinine level; and (6) positive drug screen for any substance, determined by urine drug screen at screening^100^.

### Study procedures

The study involved a total of 7 visits. The first visit was a screening visit to determine participant eligibility. The following 6 visits were sperm collection visits. During the screening visit, subjects underwent an in-office assessment including a urine toxicology screen, urine cotinine screen, and clinical assessments, including the Adverse Childhood Experiences (ACE) questionnaire, and the MINI International Neuropsychiatric Interview^100,101^. Subsequent visits (2–7) took place once a month for 6 months. At these visits, subjects submitted a semen sample, collected at home within the previous hour, to experienced andrologists at Penn Fertility Care clinic for processing and sample cryopreservation. Participants were asked to abstain from ejaculation for 48 h prior to semen collection. Within the same day, participants also completed a series of questionnaires to assess stress and anxiety experienced over the previous month, including the Perceived Stress Scale (PSS) and the Spielberger State Trait Anxiety Inventory (STAI)^61,102^. The PSS is the most commonly used instrument to assess perception of stress over the previous month^61^. It measures the degree to which situations in a subject’s life are appraised as stressful. The instrument taps into how unpredictable, uncontrollable and overloaded respondents experience their lives. The 10-item scale includes a number of direct queries about current levels of experienced stress during the last month. One participant did not return for their final donation, therefore only timepoints 1–5 were available for subject 11. In addition, one subject was excluded due to consistently low sperm quality across donated samples, leaving a final study cohort of 17 subjects for sperm sncRNA analysis.

### Sperm sncRNA sequencing

Procedures for the isolation of small RNA from mature sperm were adapted from a published protocol^103^. Briefly, cryopreserved sperm samples were thawed, suspended in PureSperm Buffer (Nidacon), then mature sperm were enriched by centrifugation (300*g*, 15 min) through a 50% PureSperm density gradient (Nidacon). Sperm were then lysed in TRIzol-LS (Thermo Fisher) reagent, supplemented with 0.2 M β-mercaptoethanol and 100 mg of nuclease-free stainless-steel beads, by homogenization on a Disruptor Genie (Scientific Industries) at 3000 rpm for 5 min. RNA, enriched for small RNA, was isolated using Qiagen’s miRNeasy Mini kit according to manufacturer’s instructions. RNA concentration and quality were assessed using Agilent’s small RNA chips run on a Bioanalyzer 2100 (Agilent Technologies). The small RNA content of sperm samples was analyzed by small RNA sequencing. Libraries were constructed using the TruSeq small RNA Library Prep Kit (Illumina). RNA input for library preparation was standardized to 10 ng of small RNA, in accordance with the manufacturer’s protocol. Post-PCR cleanup and size selection for products > 100 bp was performed using AMPure XP bead purification. Library size distribution and quantification was performed on a TapeStation 4200 (Agilent) using their High Sensitivity D1000 screentape. Individually barcoded libraries were pooled to achieve ~ 10 million reads per sample and sequenced on an Illumina NextSeq 550 (36-bp single-end).

### Bioinformatic analysis pipeline

The small non-coding RNA sequencing (sncRNASeq) analytical pipeline was designed using the snakemake framework and is available via GitHub at https://github.com/acshetty/sncRNA-seq-analysis^104^. Reference transcriptome sequences in FASTA format were downloaded from public repositories. These included the GRCh38 ncRNA reference from Ensembl, the miRNA reference from miRbase 21, the tRNA reference generated from GRCh37 by GtRNAdb 2.0, and the piRNA reference from piRBase v1.0^105–107^. Using ‘index_ref’ component, each reference FASTA file was indexed using the ‘bowtie-build’ from the Bowtie short read aligner software^108^. The sequencing reads have lengths longer than the average size of most sncRNAs which may result in the inclusion of adapter sequence at the 3′-end of the read sequence. Hence, the ‘trim_fq’ component was invoked in order to remove trailing adapter sequence using the Trimmomatic tool^109^. After trimming, reads shorter than 15 nucleotides were discarded before downstream analyses. The trimmed reads for each sample were then aligned, using the ‘align_reads’ component, to each of the different sncRNA class-specific reference transcriptomes using the Bowtie short read aligner^108^. Reads were aligned allowing for 2 mismatches and a seed length of 15 nucleotides. The alignment statistics were summarized for each sample across each of the different reference sequences using the ‘merge_alignment_statistics’ component. The raw expression values were computed using the ‘compute_expr’ component based on the number of reads aligned to each of the sncRNAs specified in their respective reference files. For each type of sncRNA, the raw expression values were merged across samples using the ‘merge_expr’ component to generate a count matrix for downstream analysis.

### Characterizing the dynamics of sperm sncRNA expression

Expression of sncRNA were adjusted for differences in library sequencing depth to generate counts per million reads (CPM) following TMM normalization using the Bioconductor package ‘edgeR’ (version 3.24.3)^110^. Features were retained for analysis if they were expressed at ≥ 1 CPM in at least 75% of samples. sncRNA aligning to each reference transcriptome were analyzed separately. Analyses were performed at the level of the individual sample, within each subject, and between subjects. At the sample level, the expression (CPM) of each feature (i.e. miRNA, tRNA, or piRNA) was ranked from highest to lowest and categorized if expressed in the top or bottom quartiles. These expression values were used to perform analyses of expression and variation at the within-subject level. Within-subject measures of expression include average expression, average ranked expression, and the number of collections a feature was expressed in the top or bottom quartile. Within-subject measures of variation included the coefficient of variation (CV) of a feature’s expression across collections, which was used to rank features from most to least variable, then categorize features if in the top or bottom quartiles of within-subject variation. Between-subject measures of expression (average expression and average rank expression) and variation [average CV expression (within-subject) and average rank CV expression (within-subject)] were calculated for each sncRNA from mean within-subject expression levels summarized across subjects.

### ‘Dynamic’ sperm sncRNA

To identify potential environmentally responsive ‘dynamic’ sncRNA, we exploited the within- and between-subjects structure of the sperm sncRNA expression data to developed screening criteria based on three a priori assumptions: (1) we assumed ‘dynamic’ sncRNA were likely to exhibit a higher degree of variation in expression over time in response to changes in the environment; (2) given the disparity in the amount of RNA present in sperm relative to ovum, we assumed sncRNA with the potential to impact offspring development would need to be highly expressed; and (3) we assumed sncRNA meeting criteria based on these first two assumptions in multiple subjects were more likely to reflect a conserved functional response to extrinsic factors^62–64^. The analysis we performed to characterize these properties and identify ‘dynamic’ sncRNA is illustrated in Fig. 1C. The first two criteria were based on within-subject expression characteristics. Features (sncRNA) were categorized as candidates in a given subject if: (1) a feature exhibited within-subject variation (CV expression) ranked in the top quartile of each class of sncRNA, and (2) the feature’s expression was ranked in the top quartile in ≥ 1 collection over the 6 months. (3) The final criteria required that a ‘dynamic’ sncRNA met the first two within-subject criteria in at least 25% (N = 5) of subjects (count candidate ≥ 5).

### Identifying ‘dynamic’ sperm sncRNA responsive to perceived stress experience

The relationship between normalized expression counts for the subsets of ‘dynamic’ sperm sncRNA and PSS scores were analyzed by implementing linear fixed effects models using the Bioconductor package ‘limma’ (version 3.38.3)^111^. We implemented 7 different models to evaluate the relationship between the expression of individual ‘dynamic’ sncRNA and PSS scores, testing the following associations: (1) miRNA ~ PSS score at the time of collection (t); (2) miRNA ~ PSS score at collection t − 1; (3) miRNA ~ PSS score at collection t − 2; (4) miRNA ~ PSS score at collection t − 3; (5) miRNA ~ (change in PSS score between t and t − 1); (6) miRNA ~ (change in PSS score between t and t − 2); (7) miRNA ~ (change in PSS score between t and t − 3). The first four of these models tested for the relationship between expression of a miRNA and PSS scores using a ‘rheostat’ approach allowing for a delayed response. The final three models tested for the relationship between miRNA expression and the change in PSS scores using a ‘delta detector’ approach. The final results were tabulated and filtered for a p-value < 0.01 and FDR < 0.2 to detect significant associations.

### Software used for data analyses

Sperm sncRNA sequencing data was trimmed, aligned, and counted using the small non-coding RNA sequencing (sncRNASeq) analytical pipeline, which is available in its entirety via GitHub at https://github.com/acshetty/sncRNA-seq-analysis. All other data processing, visualization, and statistical modeling were performed in the R software environment (version 3.5.3)^112^. Expression normalization was performed using the Bioconductor package ‘edgeR’ (version 3.24.3)^110^. Multivariate analyses were performed with the base R package ‘stats’ (version 3.5.3)^112^. Most data visualizations were generated using ‘ggplot2’ (version 3.2.0)^113^. Linear modeling of the relationships between ‘dynamic’ sncRNA expression and PSS scores was performed using the Bioconductor package ‘limma’ (version 3.38.3)^111^.

## Supplementary information

Supplementary Information.

## Data Availability

Raw and processed sequencing data are available from the Gene Expression Omnibus (GEO) database under accession code GSE159155. All other data supporting the findings of this study are available from the corresponding author upon reasonable request.
