# Supplementary material for: Repeated sampling facilitates within- and between-subject modeling of the human sperm transcriptome to identify dynamic and stress-responsive sncRNAs
Source: Sci Rep. 2020 Oct 15;10:17498. doi: 10.1038/s41598-020-73867-7 (PMC7562703; doi:10.1038/s41598-020-73867-7)
Supplement: Supplementary file 1 — Supplementary Information. [file 41598_2020_73867_MOESM1_ESM.docx]

Supplemental Materials for

**Repeated sampling facilitates within- and between-subject modeling of the human sperm transcriptome to identify dynamic and stress-responsive sncRNAs**

Christopher P Morgan^1^, Amol C Shetty^2^, Jennifer C Chan^3^, Dara S Berger^4^, Seth A Ament^2^, C Neill Epperson^5^, Tracy L Bale^1*^

**Affiliations**

^1^Department of Pharmacology and Center for Epigenetic Research in Child Health and Brain Development, University of Maryland School of Medicine, Baltimore, MD 21201, USA

^2^Institute for Genome Sciences, University of Maryland School of Medicine, Baltimore, MD 21201, USA

^3^Department of Biomedical Sciences, Perelman School of Medicine, University of Pennsylvania, Philadelphia, PA 19104, USA

^4^Department of Obstetrics and Gynecology, Division of Reproductive Endocrinology and Infertility, Perelman School of Medicine, University of Pennsylvania, Philadelphia, PA 19104, USA

^5^Department of Psychiatry, University of Colorado School of Medicine, CU-Anschutz Medical Campus, Aurora, CO

80045, USA

^*^ Corresponding author

**Corresponding author**

Tracy L. Bale, Ph.D.
Professor, Departments of Pharmacology & Psychiatry 
Director, Center for Epigenetic Research in Child Health and Brain Development
HSF3, room 9-171
University of Maryland School of Medicine
670 W. Baltimore St.
Baltimore, MD 21201

tbale@som.umaryland.edu
ph 410-706-5816

**Supplemental Table 1. Sperm sample characteristics:** Samples highlighted in red text were technical replicates or outlier samples and excluded from the final analyses. See attached

**Supplemental Figure 1. Distribution of human sperm ncRNA expression across samples.** Sequencing reads were aligned to the Ensembl reference transcriptome, filtered to retain transcripts with abundances ≥ 1 count per million reads (CPM) in at least 75% of samples, then normalized using the TMM method. The distribution of expression, in log2 CPM, across identified transcripts is displayed in a violin plot for each sample. The median expression is indicated by a black bar. This figure was generated using R (version 3.5.2) and the package ‘ggplot2’ (version 3.2.0) (<https://cran.r-project.org/>).

**Supplemental Figure 2. Multivariate analysis of human sperm ncRNA expression to identify potential outlier samples.** (**A)** Hierarchical clustering of samples based on the expression of identified ncRNA (from the Ensembl reference transcriptome) highlighted two samples, 04.01 and 06.01, as obvious outliers. In addition, the three technical replicates (Pool1, Pool2, and Pool3) clustered in a single exclusive clade. These samples correspond to libraries generated from a single RNA sample across the three rounds of library preparation and sequencing performed in this study. The clustering of these technical replicates indicated any batch effects were minimal. **(B)** Principal component analysis (PCA) clustering of sperm samples based on the expression of identified ncRNA confirmed these observations. The percentage of variation explained by the principal coordinate is indicated on the axes. In both A and B, color indicates the subject each sample was donated by. This figure was generated using R (version 3.5.2) and the package ‘ggplot2’ (version 3.2.0) (<https://cran.r-project.org/>).

**Supplemental Figure 3. Multivariate analysis of human sperm miRNA expression.** (**A)** Hierarchical clustering and **(B)** Principal component analysis (PCA) clustering of sperm samples based on the expression of 254 miRNA retained after filtering for miRNA with abundances ≥ 1 count per million reads (CPM) in at least 75% of samples. In both A and B, color indicates the subject each sample was donated by. These methods demonstrated broad clustering of samples collected from the same subject over 6 months, suggesting between-subject variation in sperm miRNA expression is greater than within-subject variation. 70% of samples (N = 69) were grouped in clusters exclusively with samples from the same subject. These clusters ranged in size from 2 to 6 samples. This figure was generated using R (version 3.5.2) and the package ‘ggplot2’ (version 3.2.0) (<https://cran.r-project.org/>).

**Supplemental Figure 4. Multivariate analysis of human sperm tRNA expression. (A)** Hierarchical clustering and **(B)** Principal component analysis (PCA) clustering of sperm samples based on the expression of 194 tRNA retained after filtering for tRNA with abundances ≥ 1 count per million reads (CPM) in at least 75% of samples. In both A and B, color indicates the subject each sample was donated by. These methods demonstrated broad clustering of samples collected from the same subject over 6 months, suggesting between-subject variation in sperm tRNA expression is greater than within-subject variation. 78% of samples (N = 76) were grouped in clusters exclusively with samples from the same subject. These clusters ranged in size from 2 to 6 samples. This figure was generated using R (version 3.5.2) and the package ‘ggplot2’ (version 3.2.0) (<https://cran.r-project.org/>).

**Supplemental Figure 5. Multivariate analysis of human sperm piRNA expression. (A)** Hierarchical clustering and **(B)** Principal component analysis (PCA) clustering of sperm samples based on the expression of 837 piRNA retained after filtering for piRNA with abundances ≥ 1 count per million reads (CPM) in at least 75% of samples. In both A and B, color indicates the subject each sample was donated by. These methods demonstrated broad clustering of samples collected from the same subject over 6 months, suggesting between-subject variation in sperm piRNA expression is greater than within-subject variation. 66% of samples (N = 65) were grouped in clusters exclusively with samples from the same subject. These clusters ranged in size from 2 to 6 samples. This figure was generated using R (version 3.5.2) and the package ‘ggplot2’ (version 3.2.0) (<https://cran.r-project.org/>).

**Supplemental Figure 6. Distribution of the number of months sperm sncRNA were expressed in top or bottom quartile for each subject.** To examine the stability of human sperm sncRNA expression rankings over time, a histogram of the distribution of the number of months each feature was expressed in the top quartile of its class in each subject is displayed for **(A)** miRNA, **(C)** tRNA, and **(E)** piRNA. These data demonstrate that sperm sncRNA expressed in the top quartile in at least one month were most likely to be highly expressed in all 6 collections from a subject across the study period. The same was not the case for features expressed in the bottom quartile of **(B)** miRNA, **(D)** tRNA, and **(F)** piRNA. This figure was generated using R (version 3.5.2) and the package ‘ggplot2’ (version 3.2.0) (<https://cran.r-project.org/>).

**Supplemental Figure 7. Class-specific expression of sperm sncRNA** **meeting within-subject criteria for ‘dynamic’ sncRNA in each subject.** We developed screening criteria to identify potential environmentally responsive ‘dynamic’ sncRNA. The first two criteria, based on within-subject measures of expression and variation, were used to screen for candidates in each subject. For a given subject, features were categorized as candidates if they exhibited within-subject variation (CV expression) ranked in the top quartile of each class of features, while also being expressed in the top quartile in at least one collection over the 6-month study period. To examine the expression patterns of these initial candidates within the context of each class of sperm sncRNA, the ranked expression (highest to lowest) of each **(A)** miRNA, **(B)** tRNA, and **(C)** piRNA was plotted across collections for each subject. The candidate sncRNA are plotted in dark blue, while all others sncRNA are plotted in light blue. Importantly, the number of sncRNA meeting these criteria varied between subjects, though 117 miRNA, 75 tRNA, and 369 piRNA met these criteria in at least one subject. This figure was generated using R (version 3.5.2) and the package ‘ggplot2’ (version 3.2.0) (<https://cran.r-project.org/>).

**Supplemental Figure 8. Overlap of sperm sncRNA meeting within-subject criteria for ‘dynamic’ sncRNA between subjects.** To identify sperm sncRNA that could be a part of a conserved response to a common environmental factor, we examined the overlap of candidate sncRNA between subjects. These data were presented in histograms displaying the count of initial candidate **(A)** miRNA, **(B)** tRNA, and **(C)** piRNA (on the Y axis) that overlapped between a given number of subjects (on the X axis). Thirty-three miRNA, 17 tRNA, and 97 piRNA met the initial criteria for ‘dynamic’ sncRNA in at least 25% of subjects (N = 5), constituting our final pool of ‘dynamic’ sperm sncRNA. This figure was generated using R (version 3.5.2) and the package ‘ggplot2’ (version 3.2.0) (<https://cran.r-project.org/>).

**Supplemental Figure 9. Relationship between perceived stress experience and the expression of the ‘dynamic’ let-7f-5p.** To assess the relationship between perceived stress experience and individual ‘dynamic’ sncRNA, we tested a series of linear models to test for relationships between sncRNA expression and total scores from the Perceived Stress Scale (PSS). We evaluated the following seven relationships for a ‘dynamic’ sncRNA’s expression level in a sperm sample and: 1) PSS score at the time it was collected (t) (PSS current time), 2) PSS score at the time of the previous collection PSS(t-1), 3) PSS score two months prior [PSS(t-2)], 4) PSS score three months prior [PSS(t-3)], 5) the change in PSS score between t and t-1 [PSS delta(t-1)], 6) the change in PSS score between t and t-2 [PSS delta(t-2)], and 7) the change in PSS score between t and t-3 [PSS delta(t-3)]. These relationships were displayed in a plot of let-7f-5p expression in log2 counts per million reads (log2 CPM) on the Y axis and the indicated derived PSS score on the X axis. This figure was generated using R (version 3.5.2) (<https://cran.r-project.org/>).

**Supplemental Figure 10. Relationship between perceived stress experience and the expression of the ‘dynamic’ miR-181a-5p.** To assess the relationship between perceived stress experience and individual ‘dynamic’ sncRNA, we tested a series of linear models to test for relationships between sncRNA expression and total scores from the Perceived Stress Scale (PSS). We evaluated the following seven relationships for a ‘dynamic’ sncRNA’s expression level in a sperm sample and: 1) PSS score at the time it was collected (t) (PSS current time), 2) PSS score at the time of the previous collection PSS(t-1), 3) PSS score two months prior [PSS(t-2)], 4) PSS score three months prior [PSS(t-3)], 5) the change in PSS score between t and t-1 [PSS delta(t-1)], 6) the change in PSS score between t and t-2 [PSS delta(t-2)], and 7) the change in PSS score between t and t-3 [PSS delta(t-3)]. These relationships were displayed in a plot of miR-181a-5p expression in log2 counts per million reads (log2 CPM) on the Y axis and the indicated derived PSS score on the X axis. This figure was generated using R (version 3.5.2) (<https://cran.r-project.org/>).

**Supplemental Figure 11. Relationship between perceived stress experience and the expression of the ‘dynamic’ miR-4454.** To assess the relationship between perceived stress experience and individual ‘dynamic’ sncRNA, we tested a series of linear models to test for relationships between sncRNA expression and total scores from the Perceived Stress Scale (PSS). We evaluated the following seven relationships for a ‘dynamic’ sncRNA’s expression level in a sperm sample and: 1) PSS score at the time it was collected (t) (PSS current time), 2) PSS score at the time of the previous collection PSS(t-1), 3) PSS score two months prior [PSS(t-2)], 4) PSS score three months prior [PSS(t-3)], 5) the change in PSS score between t and t-1 [PSS delta(t-1)], 6) the change in PSS score between t and t-2 [PSS delta(t-2)], and 7) the change in PSS score between t and t-3 [PSS delta(t-3)]. These relationships were displayed in a plot of miR-4454 expression in log2 counts per million reads (log2 CPM) on the Y axis and the indicated derived PSS score on the X axis. This figure was generated using R (version 3.5.2) (<https://cran.r-project.org/>).

**Supplemental Figure 12. Relationship between perceived stress experience and the expression of the ‘dynamic’ miR-6765-3p.** To assess the relationship between perceived stress experience and individual ‘dynamic’ sncRNA, we tested a series of linear models to test for relationships between sncRNA expression and total scores from the Perceived Stress Scale (PSS). We evaluated the following seven relationships for a ‘dynamic’ sncRNA’s expression level in a sperm sample and: 1) PSS score at the time it was collected (t) (PSS current time), 2) PSS score at the time of the previous collection PSS(t-1), 3) PSS score two months prior [PSS(t-2)], 4) PSS score three months prior [PSS(t-3)], 5) the change in PSS score between t and t-1 [PSS delta(t-1)], 6) the change in PSS score between t and t-2 [PSS delta(t-2)], and 7) the change in PSS score between t and t-3 [PSS delta(t-3)]. These relationships were displayed in a plot of miR-6765-3p expression in log2 counts per million reads (log2 CPM) on the Y axis and the indicated derived PSS score on the X axis. This figure was generated using R (version 3.5.2) (<https://cran.r-project.org/>).

**Supplemental Figure 13. Relationship between perceived stress experience and the expression of the ‘dynamic’ miR-12136.** To assess the relationship between perceived stress experience and individual ‘dynamic’ sncRNA, we tested a series of linear models to test for relationships between sncRNA expression and total scores from the Perceived Stress Scale (PSS). We evaluated the following seven relationships for a ‘dynamic’ sncRNA’s expression level in a sperm sample and: 1) PSS score at the time it was collected (t) (PSS current time), 2) PSS score at the time of the previous collection PSS(t-1), 3) PSS score two months prior [PSS(t-2)], 4) PSS score three months prior [PSS(t-3)], 5) the change in PSS score between t and t-1 [PSS delta(t-1)], 6) the change in PSS score between t and t-2 [PSS delta(t-2)], and 7) the change in PSS score between t and t-3 [PSS delta(t-3)]. These relationships were displayed in a plot of miR-12136 expression in log2 counts per million reads (log2 CPM) on the Y axis and the indicated derived PSS score on the X axis. This figure was generated using R (version 3.5.2) (<https://cran.r-project.org/>).

**Supplemental Figure 14. Relationship between perceived stress experience and the expression of the ‘dynamic’ tRNA-Gly-GCC-3-1.** To assess the relationship between perceived stress experience and individual ‘dynamic’ sncRNA, we tested a series of linear models to test for relationships between sncRNA expression and total scores from the Perceived Stress Scale (PSS). We evaluated the following seven relationships for a ‘dynamic’ sncRNA’s expression level in a sperm sample and: 1) PSS score at the time it was collected (t) (PSS current time), 2) PSS score at the time of the previous collection PSS(t-1), 3) PSS score two months prior [PSS(t-2)], 4) PSS score three months prior [PSS(t-3)], 5) the change in PSS score between t and t-1 [PSS delta(t-1)], 6) the change in PSS score between t and t-2 [PSS delta(t-2)], and 7) the change in PSS score between t and t-3 [PSS delta(t-3)]. These relationships were displayed in a plot of tRNA-Gly-GCC-3-1 expression in log2 counts per million reads (log2 CPM) on the Y axis and the indicated derived PSS score on the X axis. This figure was generated using R (version 3.5.2) (<https://cran.r-project.org/>).

**Supplemental Figure 15. Relationship between perceived stress experience and the expression of the ‘dynamic’ tRNA-Lys-CTT-1-1.** To assess the relationship between perceived stress experience and individual ‘dynamic’ sncRNA, we tested a series of linear models to test for relationships between sncRNA expression and total scores from the Perceived Stress Scale (PSS). We evaluated the following seven relationships for a ‘dynamic’ sncRNA’s expression level in a sperm sample and: 1) PSS score at the time it was collected (t) (PSS current time), 2) PSS score at the time of the previous collection PSS(t-1), 3) PSS score two months prior [PSS(t-2)], 4) PSS score three months prior [PSS(t-3)], 5) the change in PSS score between t and t-1 [PSS delta(t-1)], 6) the change in PSS score between t and t-2 [PSS delta(t-2)], and 7) the change in PSS score between t and t-3 [PSS delta(t-3)]. These relationships were displayed in a plot of tRNA-Lys-CTT-1-1 expression in log2 counts per million reads (log2 CPM) on the Y axis and the indicated derived PSS score on the X axis. This figure was generated using R (version 3.5.2) (<https://cran.r-project.org/>).

**Supplemental Figure 16. Relationship between perceived stress experience and the expression of the ‘dynamic’ tRNA-Lys-CTT-2-1.** To assess the relationship between perceived stress experience and individual ‘dynamic’ sncRNA, we tested a series of linear models to test for relationships between sncRNA expression and total scores from the Perceived Stress Scale (PSS). We evaluated the following seven relationships for a ‘dynamic’ sncRNA’s expression level in a sperm sample and: 1) PSS score at the time it was collected (t) (PSS current time), 2) PSS score at the time of the previous collection PSS(t-1), 3) PSS score two months prior [PSS(t-2)], 4) PSS score three months prior [PSS(t-3)], 5) the change in PSS score between t and t-1 [PSS delta(t-1)], 6) the change in PSS score between t and t-2 [PSS delta(t-2)], and 7) the change in PSS score between t and t-3 [PSS delta(t-3)]. These relationships were displayed in a plot of tRNA-Lys-CTT-2-1 expression in log2 counts per million reads (log2 CPM) on the Y axis and the indicated derived PSS score on the X axis. This figure was generated using R (version 3.5.2) (<https://cran.r-project.org/>).

**Supplemental Figure 17. Relationship between perceived stress experience and the expression of the ‘dynamic’ tRNA-Lys-CTT-4-1.** To assess the relationship between perceived stress experience and individual ‘dynamic’ sncRNA, we tested a series of linear models to test for relationships between sncRNA expression and total scores from the Perceived Stress Scale (PSS). We evaluated the following seven relationships for a ‘dynamic’ sncRNA’s expression level in a sperm sample and: 1) PSS score at the time it was collected (t) (PSS current time), 2) PSS score at the time of the previous collection PSS(t-1), 3) PSS score two months prior [PSS(t-2)], 4) PSS score three months prior [PSS(t-3)], 5) the change in PSS score between t and t-1 [PSS delta(t-1)], 6) the change in PSS score between t and t-2 [PSS delta(t-2)], and 7) the change in PSS score between t and t-3 [PSS delta(t-3)]. These relationships were displayed in a plot of tRNA-Lys-CTT-4-1 expression in log2 counts per million reads (log2 CPM) on the Y axis and the indicated derived PSS score on the X axis. This figure was generated using R (version 3.5.2) (<https://cran.r-project.org/>).
